# Predicting modelling for early diagnosis in early myeloproliferative neoplasms, not otherwise specified. Evidence from a machine learning study

**DOI:** 10.3389/fonc.2025.1741610

**Published:** 2026-01-26

**Authors:** Anna Scattone, Andrea Lupo, Concetta Saponaro, Margherita Sonnessa, Paolo Ditonno, Samantha Bove, Annarita Fanizzi, Giuseppe Accogli, Rossana Daprile, Francesco Alfredo Zito, Maria Colomba Comes, Raffaella Massafra

**Affiliations:** 1 Pathology Department, IRCCS Istituto Tumori “Giovanni Paolo II”, Bari, Italy; 2 Biostatistics and Bioinformatics Laboratory, IRCCS Istituto Tumori “Giovanni Paolo II”, Bari, Italy; 3 Hematology and Cell Therapy Unit, IRCCS Istituto Tumori “Giovanni Paolo II”, Viale Orazio Flacco, Bari, Italy

**Keywords:** artificial intelligence, digital pathology, machine learning, MPN-NOS, myeloproliferative neoplasms diagnosis

## Abstract

**Background:**

Recently, artificial intelligence (AI) has become a valuable tool for diagnosing and predicting outcomes in blood disorders. Whole Slide Imaging (WSI) of bone marrow biopsies (BMBs) offers detailed, high-resolution views of cells and tissues; its adoption may improve the resources dedicated to the interpretation of BMB with suspected early myeloproliferative neoplasms (MPNs).

**Methods:**

We collected a retrospective dataset of H&E-stained BMBs from 88 patients diagnosed with MPN, divided into three groups: 19 with prefibrotic primary myelofibrosis (pre-PMF), 30 with polycythemia vera (PV), and 39 with essential thrombocythemia (ET). Using AI, we framed this as a three-class classification problem. For each whole slide image, we automatically calculated cellularity and cell density. We extracted morphological features related to megakaryocytes—area, perimeter, and circularity—and summarized them for each patient using statistics like mean, standard deviation, skewness, kurtosis, and entropy. This resulted in 17 features combining nuclear morphology, cell density statistics, and cellularity. After selecting significant features with the Kruskal-Wallis test, we trained a Support Vector Machine (SVM) classifier with 5-fold cross-validation to predict MPN subtypes. We then tested the model on 13 patients with a diagnosis of MPN not otherwise specified (MPN-NOS) to assess its capacity to correctly characterize the diagnosis.

**Results:**

The model achieved a mean multiclass Area Under the Curve (AUC) of 0.70 ± 0.01 and an accuracy of 0.60 ± 0.03. When tested on the 13 patients with MPN not otherwise specified (MPN-NOS), the model agreed with pathologists’ biopsy classifications 61.5% of the time and with clinical follow-up evaluations 50% of the time.

**Conclusions:**

This study represents a first step toward the development of automated tools to support MPN diagnosis, providing potential assistance to haematologists and pathologists in the clinical management of patients.

## Introduction

Myeloproliferative neoplasms (MPNs) are clonal disorders characterised by the proliferation of cells of one or more of the myeloid lineages and the potential to support progression to myelofibrosis or acute myeloid leukaemia. They are subdivided into Philadelphia chromosome (Ph)-positive and Ph-negative MPNs. The most common Ph-negative MPNs are polycythemia vera (PV), essential thrombocythemia (ET) and primary myelofibrosis (PMF) (1–3). The 2016 WHO classification of Myeloid neoplasm refined diagnostic criteria for pre PMF to help to accurately differentiate early phase PMF and Essential Thrombocytemia (4). The recent WHO 5th edition classification also includes chronic neutrophilic leukemia (CNL), chronic eosinophilic leukemia (CEL), juvenile myelomonocytic leukemia (JMML), and myeloproliferative neoplasms, not otherwise specified (MPN-NOS) (5). For consistency with WHO terminology, in this article, we will continue to refer to these entities as MPN-NOS rather than the term *Myeloproliferative Neoplasms, Unclassifiable* (MPN-U) used in the International Consensus Classification (ICC) (6).

The latter category is defined as MPNs with clinicopathological findings that do not meet the diagnostic criteria for any other specific entity. Traditionally, the MPN-NOS spectrum includes early-phase MPNs, overlapping clinicopathological conditions, advanced fibrotic MPNs, cases associated with inflammatory or neoplastic disorders that obscure the clinical and histological picture, and poorly characterized MPNs with clinicopathological discrepancies. The diagnosis of MPN relies on established criteria, which are primarily based on clinical and laboratory parameters, molecular diagnostics (mutually exclusive mutations in specific areas of the following genes: Janus kinase 2 - JAK2, calreticulin - CALR, or the thrombopoietin receptor/myeloproliferative leukemia gene - MPL), and the morphological assessment of bone marrow biopsy (5–7). In most cases, diagnosis occurs during the chronic phase (CP), although progression to an acute phase (typically with myeloid differentiation) is part of the natural course of the disease. However, some cases present with MPN-like features that do not fully meet the established diagnostic criteria for a specific MPN subtype or exhibit overlapping characteristics. Such cases are classified as myeloproliferative neoplasms, not otherwise specified (MPN-NOS) [according to WHO 2022; unclassifiable (MPN-U) according to ICC 2022] (2, 8). In the early phases of MPN, overlapping clinical features can complicate the assignment of an accurate diagnosis. Early phase Primary Myelofibrosis, Masked Polycythemia Vera and early Essential Thrombocythemia are clear examples where distinguishing between subtypes can be particularly challenging. The therapeutic approach is generally tailored to the specific MPN diagnosis and considers risk stratification. It can range from watchful waiting to allogeneic stem cell transplantation. Accurate disease assessment is therefore crucial to guide the patient towards the most appropriate therapeutic pathway. However, an MPN-NOS diagnosis leaves the patient in a position where treatment decisions may lack the support of robust scientific evidence. As a result, developing new methods for achieving a definitive and precise diagnosis is becoming increasingly important, especially for MPN-NOS. In recent years, the analysis of biomedical images through artificial intelligence (AI) has revealed a powerful resource for the diagnosis and prognosis of diseases, including both solid tumors and hematologic disorders (9, 10). Whole Slide Images (WSIs) representing Bone Marrow Biopsies (BMB) provide a high-resolution representation of cellular structures and marrow microenvironment, thus enabling a potential detailed analysis of the tissue for MPN diagnosis (11). However, this kind of analysis is challenging since the interpretation of WSIs requires specialised expertise and could be subject to inter-observer variability. Moreover, the increasing volume of data generated by modern imaging technologies makes the automation of the analysis process essential to improve efficiency and consistency of diagnosis (12).

Major criteria dictated by WHO to recognize a myeloproliferative neoplasm are the bone marrow cellularity and the megakaryocytic proliferation and atypia (5). Pathologists primarily focus on variations in cell size, atypia (e.g., nuclear lobulation/complexity), clustering of megakaryocytes, along with the number and shape of these clusters, as key factors in identifying MPN. MPN diagnosis prediction by means of AI-based models is an active research field, which has been freshly investigated with promising results (13). Some efforts have focused on the automated-based identification of megakaryocytes: Sirinukunwattana K. et al. (9) describes a novel computational method for the systematic analysis of tissue megakaryocytes. This incorporates a platform that combines manual annotation tools with support from AI models for the automated-based identification of megakaryocytes. It assists hematopathologists in the efficient identification of megakaryocytes from routinely prepared slides. However, there is a lack of research work investigating the prediction of MPN diagnosis (PV, ET, PMF) by AI models exploiting quantitative imaging information automatically extracted from WSIs representing BMB slides.

Within this emerging scenario, the present study aims to explore the application of AI to WSIs for the prediction of MPN diagnosis. As far as we know, only a limited number of studies have investigated AI-based approaches for the automated classification of MPNs from bone marrow biopsies. In particular, recent work has explored deep-learning models, including convolutional neural networks and attention mechanisms, applied directly to whole-slide images to capture megakaryocyte morphology and spatial context for MPN classification (14, 15).Our work proposes a fully automated AI-based pipeline to firstly automatically extract morphological features from the BMB slides under study, which were then exploited by a classifier to discern the three most common MPN subtypes (focusing on the early phase of Primary Myelofibrosis): PV, ET, pre-PMF. We also validated the model on a set of 13 MPN-NOS patients, that are patients for which the diagnosis was non-specific and compared the prediction of the model with the classifications performed by pathologists at the time of biopsy, as well as by clinicians at follow-up.

This research work represents a first effort towards the development of an automated decision support tool to enable a faster, more accurate and reproducible MPN diagnosis, thus facilitating optimal clinical management of patients affected by these diseases.

## Materials and methods

### Data collection and problem formulation

A retrospective series of 101 consecutive BMBs of patients with MPNs were included in this study and collected from 2011 to 2023. The patients were selected on the basis of diagnosis of essential thrombocythemia (ET), polycythaemia vera (PV), prefibrotic myelofibrosis (pre-PMF) and myeloproliferative neoplasm, not otherwise specified (MPN-NOS), reported at the Pathological Anatomy Unit of the Istituto Tumori “Giovanni Paolo II” of Bari, Italy. Two experienced pathologists reviewed all the slides at a multi-headed microscope. They discussed the discrepant cases, but especially to specify so-called MPN-unclassifiable (MPN-U) cases to reach a univocal concordance of diagnosis.

The diagnosis was made according to the criteria of 5^th^ edition of the WHO, evaluating bone marrow histological examination, clinical and laboratory data and the driver mutation genetic tests (JAK2, CALR and MPL). The study was approved by the Ethics Committee of the Istituto Tumori “Giovanni Paolo II” with document n. 1309/CE of September 13, 2023. The cases were recruited from the Histological archive of the Pathological Anatomy Unit of our Institute on the basis of the diagnosis and availability of histological material. The JAK2 V617F mutation located in exon 14 of the JAK2 gene was detected using real-time PCR (qPCR) and the commercial kit ipsogen^®^ JAK2 MutaQuant^®^ from QIAGEN (Hilden, Germany). This is a quantitative *in vitro* assay for the accurate detection and quantification of the JAK2 V617F/G1849T. The absence of this mutation does not exclude the presence of other JAK2 mutations, in nucleotides from 88504 to 88622. This test is based on the principle of hydrolysis of double fluorochrome oligonucleotide quantitative Real Time PCR (qPCR) performed on the Rotor-Gene Q MDx 5plex HRM instrument. The MPL W515L and MPL W515K mutations were detected using the commercial ipsogen^®^ [inserire specifiche kit] MPL W515L/K MutaScreen Kit from QIAGEN (Hilden, Germany), following the manufacturer’s instructions on Rotor-Gene Q 5 plex HRM instrument. For the CALR gene, the two major CALR mutations (Type 1 and Type 2) were identified with the ipsogen^®^CALR RGQ PCR kit from QIAGEN (Hilden, Germany) on Rotor-Gene^®^ Q MDx 5plex HRM instrument, in association with the Rotor-Gene AssayManager v2.1 software for automated interpretation of results. This kit is a Real-time PCR test, that uses quantitative Realtime PCR (qPCR) techniques for the qualitative detection of somatic mutations in the c.1091_1162 region (cDNA annotation) of exon 9 in the CALR gene to allow the identification of the two main CALR mutations (Type 1 and Type 2). To resolve inconsistencies, NGS was employed to investigate JAK2, CALR, and MPL mutations in a peripheral blood sample.

The clinical features referred to the dataset are described in the **Table 1**, categorized by each MPN disease subtype and the MPN-NOS class. Patients are distributed in the classes as follows: 19 pre-PMF, 30 PV, 39 ET and 13 MPN-NOS. Among the clinical features highlighted in the **Table 1**, the continuous characteristics are: Hb (the haemoglobin value), HCT (the haematocrit value, t), Cellularity (the quantitative determination of the cellular component in a BMB slide as a percentage) and the Age at diagnosis. They are all expressed as the mean value and standard deviation. The categorical features are: Jak2_Mutation (mutation of Janus kinase 2 gene), CALR_mutation (mutations in the gene for calreticulin), MLPW515_mutation (mutation in *MPL* gene), JAK2+CARL_mutation, MF (semiquantitative bone marrow fibrosis) and Sex. The mutations are expressed as the number of patients (also in percentages) with mutation/fibrosis (Presence), without mutation/fibrosis (Absence). MF (myelofibrosis) was evaluated adopting the semiquantitative WHO grading system (on a scale of MF-0 to MF-3). The last feature is expressed as the number of female and male patients (also in percentages) for each subtype.

**Table 1:** Distribution of clinical features referred to the dataset for each subtype disease.

Characteristics	Pre-PMF	PV	ET	MPN-NOS
JAK2_MUTATION				
*Presence*	12.00 (63%)	30.00 (100%)	26.00 (67%)	11.00 (85%)
*Absence*	7.00 (37%)	0.00 (0%)	13.00 (33%)	2.00 (15%)
CALR_MUTATION				
*Presence*	5.00 (26%)	0 (0%)	12.00 (31%)	2.00 (15%)
*Absence*	14.00 (74%)	30.00 (100%)	27.00 (69%)	11.00 (85%)
MLPW515_MUTATION				
*Presence*	1.00 (5%)	0 (0%)	1.00 (2%)	0.00 (100%)
*Absence*	18.00 (95%)	30.00 (100%)	38.00 (98%)	13.00 (100%)
JAK2+CARL_MUTATION				
*Presence*	1 (5%)	0 (0%)	0 (0%)	0 (0%)
*Absence*	18 (95%)	30 (100%)	39 (100%)	13 (100%)
HCT *Mean_value* ± *Dev_std*	41.95 ± 8.30	51.85 ± 7.00	42.90 ± 4.20	45.00 ± 6.88
Cellularity *Mean_value* ± *Dev_std*	0.55± 0.12	0.67 ± 0.13	0.65 ± 0.14	0.63 ± 0.13
MF				
*0*	1.00 (5.27%)	3 (10%)	12.00 (30.77%)	2.00 (15.38%)
*1*	18 (94.73%)	27 (90%)	27.00 (69.23%)	11.00 (84.60%)
Sex				
*F*	13.00 (68%)	17.00 (56.67%)	19.00 (49%)	8.00 (62%)
*M*	6.00 (32%)	13.00 (43.33%)	20.00 (51%)	5.00 (38%)
Age at diagnosis *Mean_vaue* ± *std*	66.00 ± 13.60	64.00 ± 13.51	53.00 ± 15.41	61.00 ± 10.34
WB				
*Mean_vaue[x10^3/µl]* ± *std*	10.1 ± 3.85	12.2 ± 6.08	7.8 ± 1.35	9.7 ± 2.53
PLT				
*Mean_vaue[x10^3/µl]* ± *std*	761.7 ± 291.50	613.2 ± 290.36	662.2 ± 183.01	618.7 ± 185.50
Hb				
*Mean_vaue[g/dl]* ± *std*	13.1 ± 1.89	17.1 ± 0.99	12.9 ± 0.99	14.8 ± 1.43

For categorical characteristics, counts and percentages are reported; for continuous features mean value and standard deviation are specified.

### Immunohistochemistry and digitalization

3-micron (µm) sections from histological samples, Paraffin-Embedded, have been cut and stained using hematoxylin-eosin (H&E). BM fibrosis (MF) was assessed according to the WHO grading system (5).

The H&E-stained tissue section is the capstone of anatomical pathology diagnosis. This technique is realised using the Tissue-Tek Prisma Plus automated staining instrument (Sakura) with Tissue-Tek^®^ H&E Staining Kit. All reagents of the Hematoxylin and Eosin Kit, used according to factory-validated staining protocol, provided with the kit, are ready-to-use in bottles. Digital slides were obtained by using a high-performing slide scanner at 40×magnifcation (Aperio AT2, Leica Biosystems).

The collected BMB slides were used to solve a three-class classification problem. In detail, we firstly automatically extracted morphological features for each digitised image and then, we used them to train an AI-based model to predict the MPN subtype (PV, ET, pre-PMF). For each BMB slide, the image analysis described in the following sub-paragraphs was performed on three Regions Of Interest (ROIs), which were selected by two expert pathologists of our Institute in mutual agreement. These ROIs nearly cover the entire slide, selecting areas that contain the intertrabecular spaces with hemopoietic cells, avoiding regions with tissue folds, tears, red blood cells, out-of-focus areas, air bubbles, excessive background, or extreme staining variations to ensure image quality and diagnostic interpretability and with lymphoid aggregates. The primary purpose of using two expert pathologists was indeed to establish a robust “consensus ground truth”. This approach aims to minimize noise from individual subjective assessments.

### Automatic cellularity computation

Cell count is a key criterion established by the WHO for disease diagnosis (5).Thus, quantifying cellularity is essential for pathologists when assessing MPN subtypes. Cellularity refers to the quantitative measurement of the cellular component in a BMB slide and can be classified as normal, reduced, or increased. In clinical practice, pathologists estimate cellularity according to specific guidelines and empirical assessment; the marrow is classified as hypoplastic or hyperplastic when the hematopoietic component of the sample is respectively below or above the normal range expected for the patient’s age (16). However, this method is subject to variability between operators.

**Figure 1:**
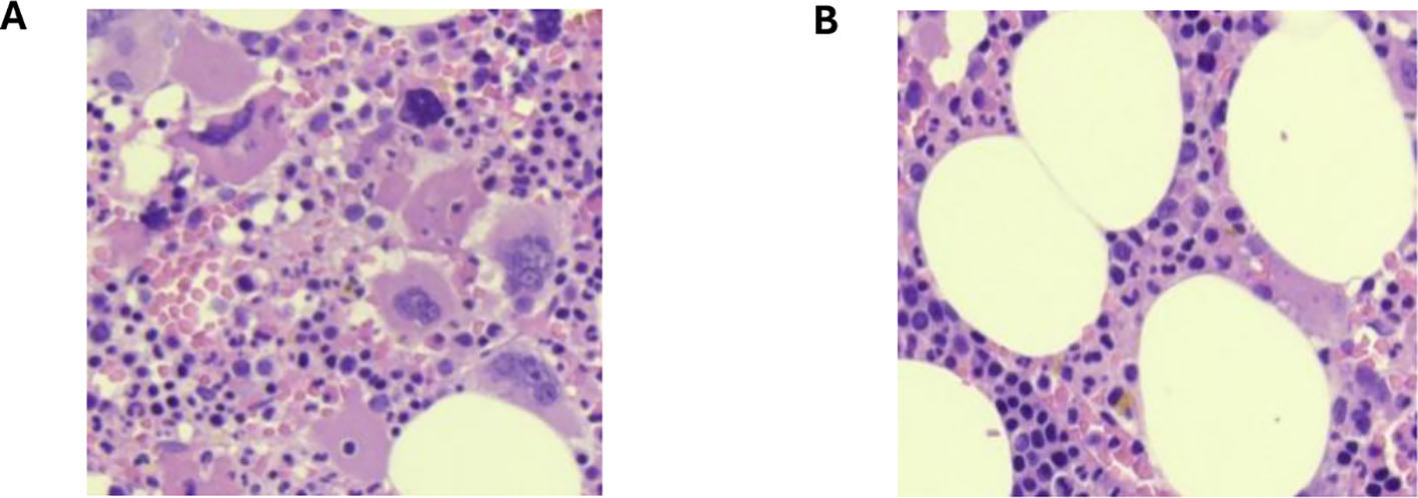
Cell population within a BMB slide. **(A)** Nucleated cells: erythroid series and megakaryocytes. **(B)** Non-nucleated cells: adipocytes.

To minimize this variability, we developed an automated, computer-based method to measure cellularity in BMB slides. This method provides an objective evaluation of the proportion of hematopoietic components in a bone marrow sample, providing essential support to pathologists in their analysis. We define cellularity as the ratio of the area occupied by nucleated cells (including myeloid and erythroid series, as well as megakaryocytes in the case of BMB slides, see **Figure 1A**) to the total area examined, which includes both nucleated and non-nucleated cells. In our analysis, the non-nucleated cells were identified as adipocytes (see **Figure 1B**).

Cellularity can be expressed with the following formula (17):


$$Cellularity=\frac{area\;cells\;with\;nucleus}{\left(cells\;with\;nucleus+cells\;without\;nucleus\right)area}$$

**Figure 2:**
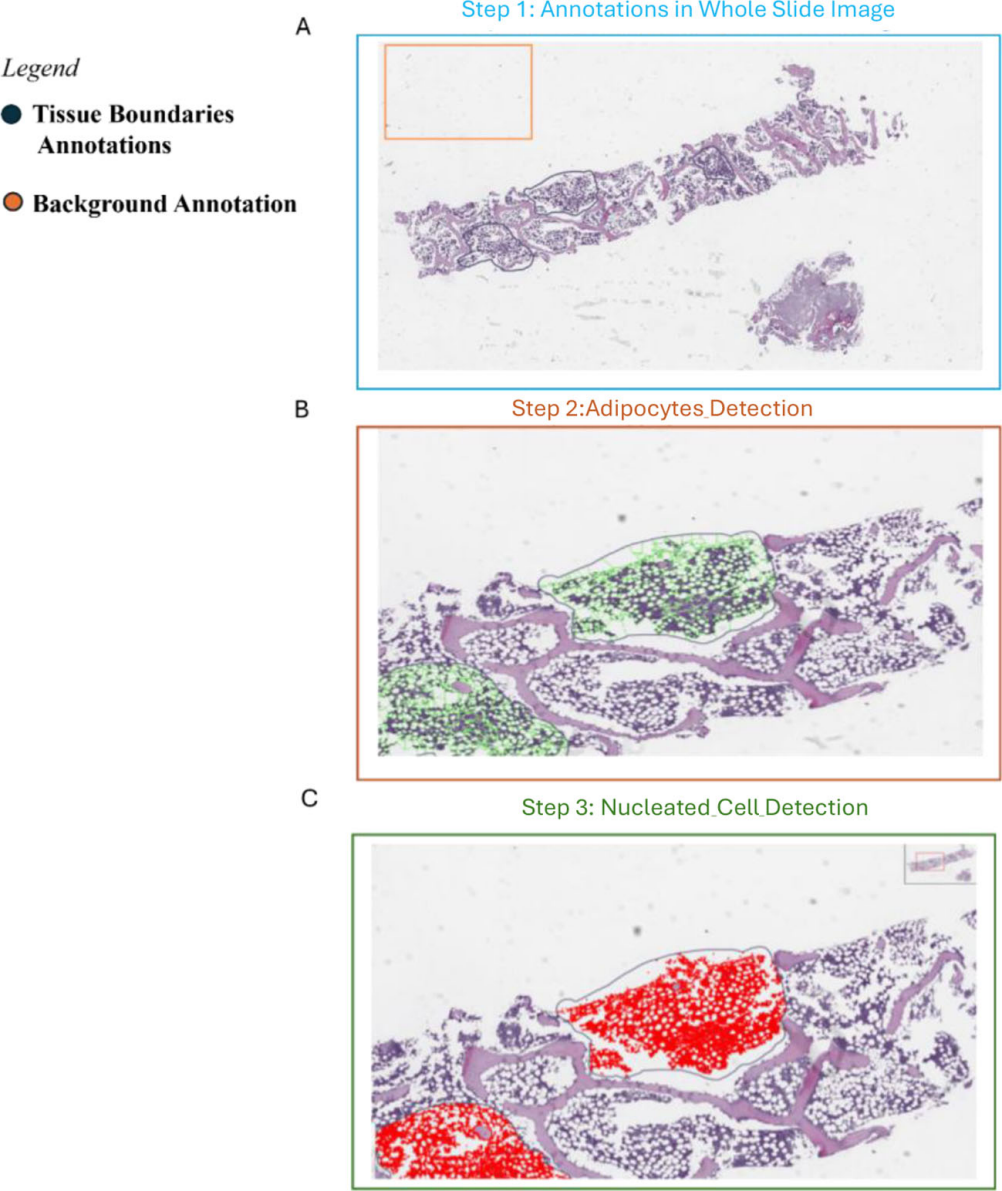
Workflow of the proposed automatic cellularity computation algorithm.

We developed an automatic detection algorithm to compute both nucleated cells and adipocytes, revising the detection algorithm proposed by Tratwal J. et al. (17). The algorithm was implemented in QuPath software (18) and consisted of three steps:

Step 1- *Annotation* (**Figure 2A**): Two annotation classes were predefined for each BMB slide: *Tissue Boundaries* and *Background*. The Tissue Boundaries define the ROIs containing hematopoietic tissue to analyze. The Background class helps correct for background color across the whole slide and allows the algorithm to differentiate between the white background and the white areas representing adipocytes. During sample preparation, fat cells dissolve and create empty spaces with pixel values similar to the background. The algorithm compares pixel values in the ROI to those in the background to identify adipocyte regions.
Step 2- *Adypocytes detection* (**Figure 2B**): We binarized the ROI areas of the BMB slide, then applied the *Watershed algorithm* to separate cell boundaries by interpreting the image as a landscape of peaks and valleys. Afterwards, the adipocyte image was dilated using ImageJ within QuPath, with 50 iterations repeated 5 times. Fixed parameters for adipocyte detection included minimum circularity of 0.3 and minimum size of 5 pixels to identify adipocytes accurately.
Step 3- *Nucleated cell detection* (**Figure 2C**): Nucleated cells were automatically detected using *WatershedCellDetection* plugin in QuPath with standard settings: pixel size of 0.5 µm, background radius 8.0 µm, median filter radius 0 µm, Gaussian sigma 1.5 µm, minimum and maximum cell areas of 10 and 400 µm², intensity threshold 0.1, maximum background intensity 2.0, and cell expansion of 5.0 µm. The Watershed algorithm was also applied to binarized images to refine cell boundaries.

Finally, we computed the automatic cellularity to be added to the set of the other morphological features automatically extracted from the ROIs to solve the classification task.

### Morphological feature extraction

By using the QuPath function *saveDetectionMeasurements*, we automatically extracted some morphological features related to nuclei of the nucleated cells detected within the three ROIs of each slide: area, perimeter, circularity. We focused on nuclei with eccentricity greater than 0.8 and maximum caliper greater than 20 to identify megakaryocytes, which are key for MPN diagnosis due to their larger nuclear size (19). An increase in their nuclear area is also an important pathological indicator (5). For each of the nucleated cells, these three characteristics were extracted. To analyze variation across cells, we computed five statistics for each feature per patient: mean, standard deviation, skewness, kurtosis, and entropy, resulting in 12 features per patient.

Additionally, cell density was calculated for each ROI to quantify the presence of megakaryocytes relative to smaller-nucleated cells (erythroid series). Cell density was defined as the ratio of the total megakaryocyte nuclear area to the total nuclear area of all cells. The values from the three ROIs were summarized using the same five statistics.

Including the automatic cellularity measure, we compiled a set of 17 features per patient: statistics for each nuclear feature, statistics for cell density, and cellularity.

### Feature selection and classification

Firstly, the association between each feature and MPN subtype (PV, ET, pre-PMF) was evaluated by means of the nonparametric Kruskal-Wallis test (20). Only features with a p-value less than 0.05 were selected, standardized, and used as input for a Support Vector Machine (SVM) with a linear kernel (21) to predict the MPN subtypes for each patient. A SVM with a linear kernel was chosen because it is well suited to relatively small datasets with high-dimensional, handcrafted feature spaces and preserves the biological interpretability of the input variables. The classifier was evaluated using a five-fold cross-validation scheme repeated five times, ensuring that, in each fold, the test set remained completely independent from the training set and that all patients contributed to the independent test set across repetitions.

The predictive performances of the classifier were evaluated in terms of the Area Under the Curve (AUC) for a multiclass classification problem (22), which is defined as the sum of the weighted AUC for each label:


$$AUC_{tot}=AUC_{PMF}\times weight_{PMF}+AUC_{PV}\times weight_{PV}+AUC_{TE}\times weight_{TE}$$

Each weighted AUC was computed as the product between the label weight and the label AUC, where the label weight is the number of patients belonging to the class identified by that label on the total number of patients, while the label AUC is the well-known *One vs Rest value*, which was computed by comparing the class referred to that label against all the other classes put together. For the AUC value computation, we take the class of reference (one class) was considered as the “positive” class, while all the other classes (the rest) are considered as the “negative” class. The predictive performances were also evaluated in terms of the total accuracy for a multiclass classification problem, which is expressed as the ratio between the correct predictions over the total number of samples under study.

Finally, we evaluated the Micro F1-score and the Micro G-mean for a multiclass classification problem. We firstly computed the Sensitivity, Specificity and the Precision for each round and we considered the mean as the final predicted performance.


Sensitivity= ∑​tp/(∑​tp+∑​fn)

Specificity= ∑​tn/(∑​tn+∑​fp)

Precision= ∑​tp/(∑​tp+∑​fp)

where tp are the True Positive, the really positive examples, classified as these from our artificial intelligence model; tn are the True Negative, the really negative examples, predicted as these from our model. On the other side, fp and fn are False Positive and False Negative respectively: fp are the really negative examples, classified as positive from our model and consequently fn are the really positive, predicted as negative from our model.

The Micro F1-score and the Micro Geometric Mean were expressed by the following formulas:


Micro F1−SCORE= 2*( Sensitivity*Precision)/(Sensitivity+Precision)

$$Micro\;G-mean=\sqrt{Sensitivity\;*Specificity}\;$$

Finally, we validated the classifier on 13 MPN-NOS patients, whose diagnoses were not classified as PV, ET, or pre-PMF. The model was trained on patients from these three subtypes, and each MPN-NOS patient was assigned to the subtype with the highest classification score. These predictions were compared with the pathologists’ and clinicians’ assessments at biopsy and follow-up. Machine learning simulations and model training were conducted in MATLAB R2024a (MathWorks, Natick, MA, USA).

## Results

### Characterization of the validation cohort (MPN-NOS)

We identified 13 cases of MPN-NOS that did not fulfil the diagnostic criteria for any specific entity in accordance with WHO 2022 classification, along with one case exhibiting uncanonical molecular features. The median age at diagnosis was 61 years (range: 43–84), with a slight female predominance (8/13, 62%). Laboratory data: lactate dehydrogenase (LDH) was elevated in 2 out of 13 patients (16.6%); thrombocytosis was observed in 12 out of 13 patients (92.3%), with a median platelet count of 618.7 × 10^9/L; elevated Hb levels and WBC counts were present in 3 out of 13 patients (23%) with a median WBC count of 9.8 × 10^9/L and a mean hb value of 14.8 g/dL. Mild splenomegaly was documented in 2 out of 13 patients (15.3%). On a molecular level, the JAK2V617F mutation was detected in 11 out of 13 patients (84.6%), while 2 out of 13 (15.4%) had CALR mutations. One case showed a CALR type 1 mutation, while the second displayed a distinct frameshift mutation in the CALR gene located on exon 9, defined as del 9c.1121_1139del; p.(Lys374Argfs*) with a variant allele frequency (VAF) of 40.4%.

Two expert pathologists classified the MPN-NOS cases into three morphological subgroups and evaluated the clinical features. According to WHO-HEM5 criteria, they found that in the cluster of patients (five cases) with an essential thrombocythemia-like (ET-like) morphology (BMB15, BMB 93, BMB 1643, BMB 1644, BMB 79), characterized by normocellular or mildly hypercellular marrow and a minor increase in megakaryocytes, clinical features were discrepant in two cases. In BMB 15 and BMB 93, the patients presented only mild erythrocytosis (>16.5 g/dL, meeting the first major diagnostic criterion in the WHO-HEM5), but with normal erythropoietin (EPO) levels. In follow-up, the disease was reclassified as polycythemia vera (PV) in both cases due to the occurrence of EPO levels below local laboratory ranges (4.3–29 mU/mL). In BMB 1643 and BMB 1644, an overlapping morphology between ET and Pre-PMF was observed. BMB 1643 lacked evident atypia of megakaryocytes, but showed slightly hypercellular marrow and increased reticulin fibrosis, which contrasted with ET. In follow-up, the disease developed into pre-PMF, as documented by a subsequent bone marrow biopsy. BMB 1644 showed a morphological analysis indicative of mildly hypercellular marrow, left-shifted granulopoiesis, and increased mature-looking megakaryocytes, some of which were atypical. At onset, driver mutations of JAK2, CALR, and MPL were not detected, and a diagnosis of triple-negative (TN) MPN was made. Upon follow-up, a distinct CALR variant mutation (a frameshift mutation on exon 9c.1121_1139del) was detected using NGS, although the disease remained clinically and histologically unchanged.

The histological examination of sample BMB 79 did not robustly support the clinical diagnosis of essential thrombocythemia (ET). While molecular testing using quantitative PCR revealed a low burden of the JAK2V617F mutation, the absence of fully developed morphological features in the tissue suggests that the patient may be in an early phase of MPNs.

The cluster of patients with polycythemia vera-like morphology (PV-like) consisted of 4 cases (BMB 103, BMB 36, BMB 63, BMB 40). In BMB 103, the patient exhibited erythrocytosis (>16.5 g/dL, meeting the first major diagnostic criterion defined in WHO-2022); as EPO levels lowered in the follow-up, the disease was reclassified as PV, meeting the minor diagnostic criteria defined by WHO 2022 (EPO levels below local laboratory ranges). In BMB 36, the case displayed overlapping morphology between PV and Pre-PMF and a discrepant clinical phenotype of PV; however, the disease remained unchanged upon follow-up. BMB 63 had a polycythemia vera-like morphology but did not meet the first major diagnostic criteria according to WHO 2022. Over time, the patient reported symptoms of hemorrhoids with bleeding, suggesting features masked as PV. BMB 40 exhibited PV-like morphology but lacked the first major diagnostic criteria per WHO 2022; follow-up data were not available.

In the final cluster of patients with primary myelofibrosis-like morphology (PMF-like) (BMB 2, BMB 24, BMB 114, BMB 41), BMB 2 showed hypercellular marrow with increased granulopoiesis, with atypia of megakaryocytes, and an increase in reticulin fiber (MF1). In this case, distinguishing between ET and Pre-PMF was challenging. Notably, during close follow-up, the patient developed synchronous breast and lung cancers, leading to reclassification as ET according to WHO 2022 diagnostic criteria. BMB 24 exhibited overlapping morphology between ET and Pre-PMF, characterized by increased granulopoiesis and reticulin fiber (MF1), as well as some degree of megakaryocyte atypia. This case also presented a discrepant clinical phenotype mimicking ET, contrasted by elevated LDH levels upon follow-up. BMB 41 and BMB 114 displayed overlapping morphology between ET and Pre-PMF, with hypercellular marrow, increased granulopoiesis without evident megakaryocyte atypia, increased reticulin fiber (MF1), and elevated LDH levels. Follow-up revealed that the disease remained unchanged, with only elevated LDH levels.

In summary, 6 out of 13 cases (46.2%) were reclassified into specific entities upon follow-up. Most cases exhibited hypercellular bone marrow with normal erythropoiesis and increased granulopoiesis, often with left-shifting (less evident in the ET-like cluster). Notably, loose megakaryocyte clusters were present in most cases, while dense and polymorphic clusters were identified in approximately 15.3% of the bone marrow biopsies. Megakaryocytes frequently displayed giant forms, hyperlobulated or bulbous nuclei, and maturation defects. Interestingly, almost all cases were in the prefibrotic stage (MF1) according to WHO criteria.

### Statistical analysis results

We used the Kruskal-Wallis test to evaluate the association between each feature and the MPN subtypes (PV, ET, pre-PMF). Six features showed statistically significant differences (p-value< 0.05). Three were related to the cell nucleus: the mean and skewness of the nuclear perimeter (p = 0.0423 and 0.0104) and the skewness of nuclear circularity (p = 0.0474). Two features described cell density: the mean (p = 0.0329) and skewness (p = 0.0384). The last significant feature was the automatic cellularity value (p = 0.0000772).

**Figure 3:**
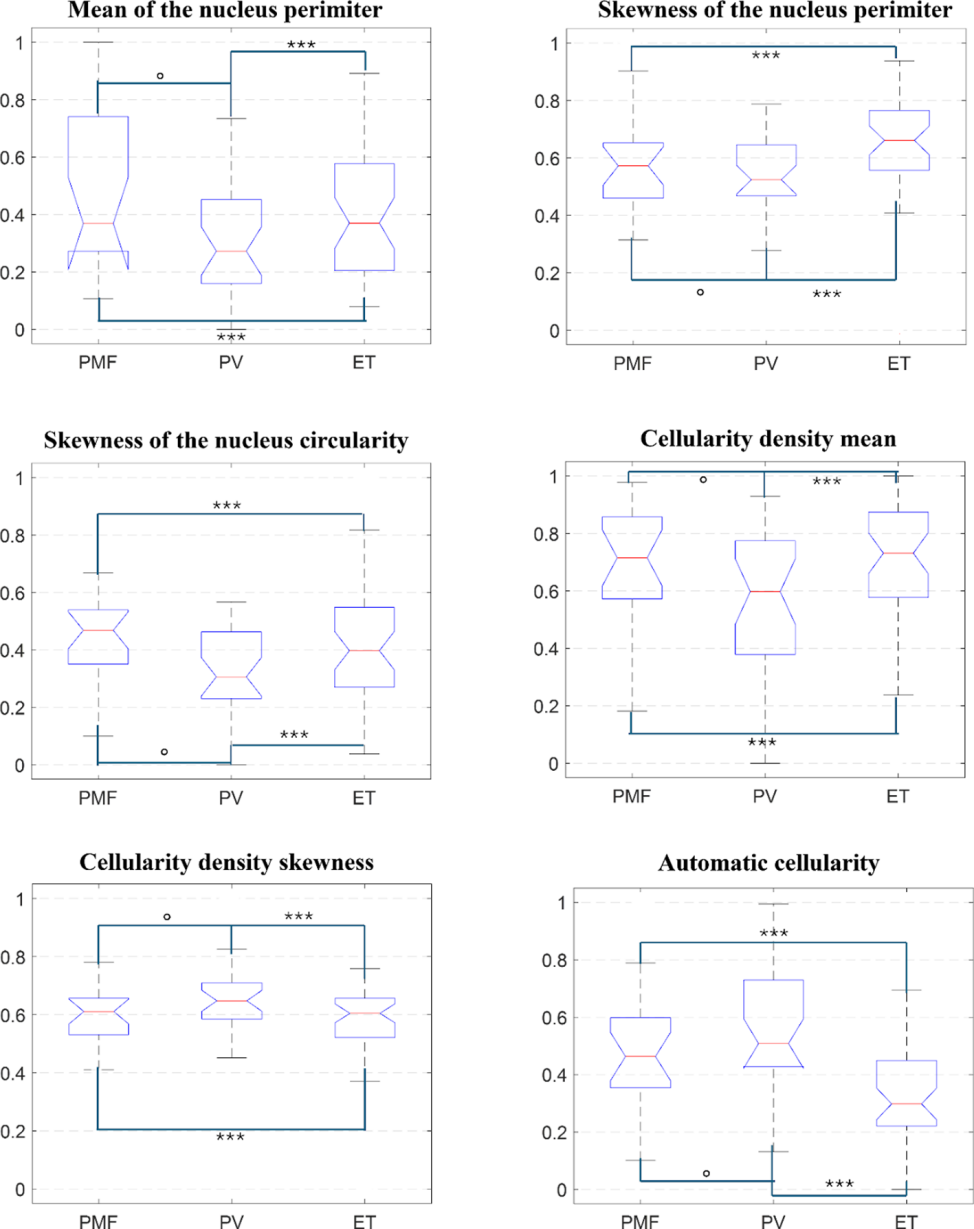
Kruskal-Wallis Boxplots related to the six significant features. For each feature, the distributions were compared all together or in pairs. “o” indicates p-value > 0.05, while “***” stands for p-value< 0.001.

**Figure 3** shows boxplots of these features’ distributions across the classes. We also performed pairwise comparisons between subtypes and found significant differences between pre-PMF and ET, and between PV and ET, for all significant features (p = 0.0008). This indicates these features help distinguish these MPN subtypes.

### Comparison between automatic and pathologists’ cellularity

We validated our automatic cellularity measurement by comparing it with values provided by expert pathologists for each bone marrow biopsy slide. The pathologists’ data included exact percentages and percentage ranges; the upper bound of the range was used as the reference.

To compare the two sets, we applied the two-sample Kolmogorov-Smirnov (K-S) test, which compares the distributions of two samples. Our test returned a p-value of 0.39, indicating no significant difference between the automatic and clinical cellularity distributions. The average absolute difference between the two measurements was approximately 0.1 to 0.2.

**Figure 4:**
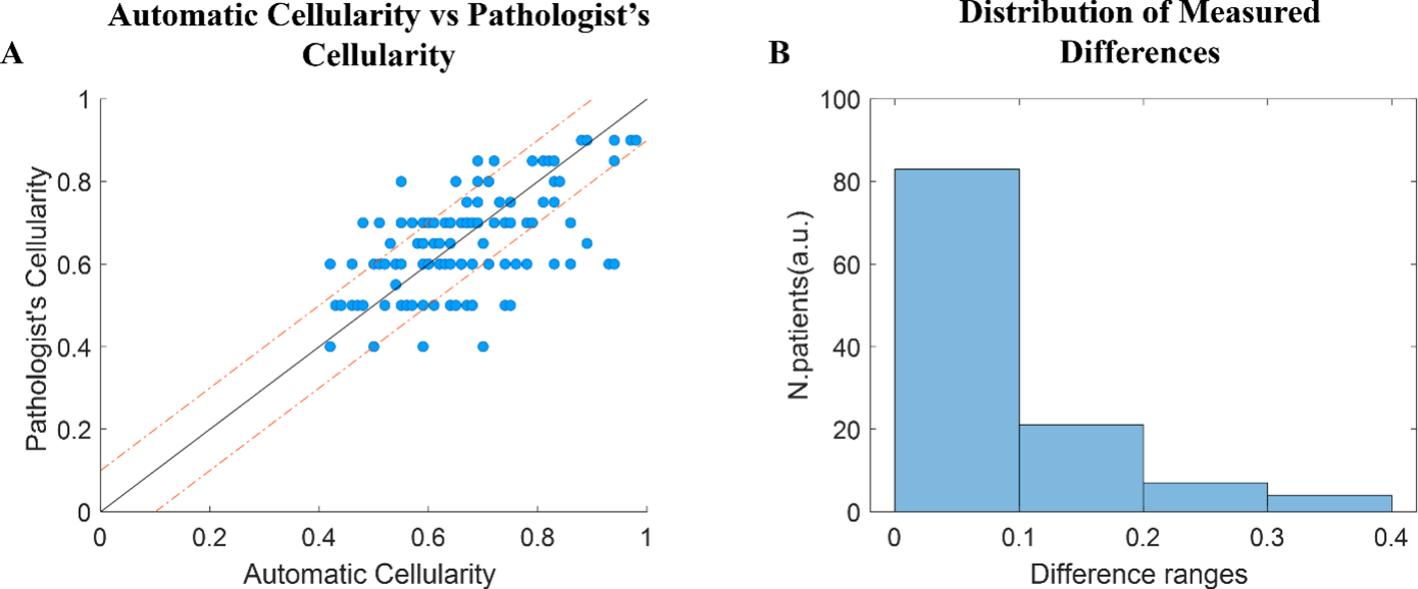
Automatic cellularity values against clinical cellularity values. **(A)** Scatter plot of differences between d pathologist’s values and automatically calculated values for each patient. **(B)** Histogram of distribution of the measured differences in specific intervals.

To visualize the differences, we used a scatter plot highlighting patients with differences greater than 0.1, and a histogram displaying the distribution of differences in intervals of 0.1 from 0 to 0.4. Most differences fell within 0–0.1 and 0.1–0.2 intervals, showing that the automatic and pathologist measurements mostly agree (see **Figure 4**).

### Classification results

**Figure 5:**
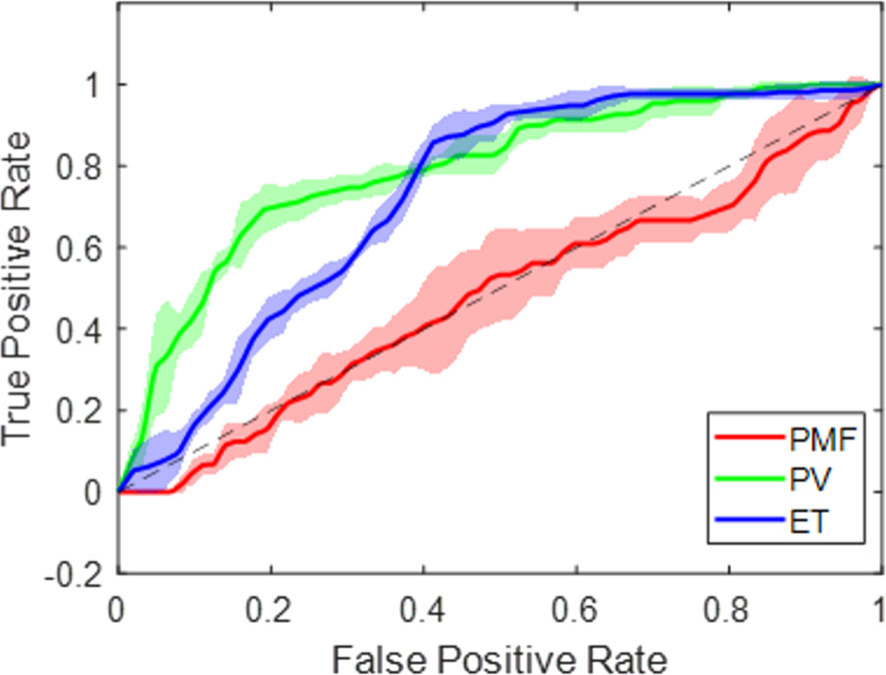
ROC curves computed for each class according to the rationale *One vs Rest*. The ROC curves stand for mean curves. The shaded areas represent standard deviations. The mean and standard deviation of x- and y-coordinates were computed over all the folds and rounds. The dashed line indicates random guess.

The classifier was firstly validated on the dataset of 88 patients at disposal with a confirmed diagnosis, using a five-fold cross-validation repeated five times. In each fold, the test set was kept independent and subsequently aggregated to obtain the final validation cohort. The mean multiclass AUC value was 0.70 ± 0.01, where the mean and standard deviation values were computed over both the folds and rounds. The mean multiclass accuracy value was 0.60 ± 0.03, computed as the mean value over all the rounds. The mean AUC values of each class were 0.50 ± 0.04, 0.80 ± 0.03, and 0.73 ± 0.06 for pre-PMF, PV and ET classes, respectively. The lower AUC observed for the pre-PMF class reflects the limited number of available cases. Although class-weighted SVM models were explored to compensate for class imbalance, these strategies did not lead to stable or meaningful improvements in performance and were therefore not retained. Consequently, class imbalance was primarily addressed at the evaluation stage by adopting metrics more robust to unequal class frequencies, including weighted AUC, micro-averaged F1-score, and G-mean. **Figure 5** shows the corresponding average ROC curves with confidence intervals for each class label. The Pre-PMF confidence interval is the largest one, because of the lower precision in estimation. The micro F1-SCORE and micro G-mean values were 0.50 and 0.60, respectively.

Finally, our automated pipeline was validated on the dataset including 13 MPN-NOS patients. Each patient was classified into one of the three classes based on the highest score obtained. **Table 2** lists the 13 MPN-NOS patients alongside the classification scores for each class and the final prediction made by our model. These predictions were compared with classifications performed by pathologists at the time of biopsy (timing 0), as well as follow-up. Out of the 13 cases in the table (2), 8 predictions align with the initial histological classification. This represents a 61.5% concordance rate between the model’s predictions and the histological assessment made at the time of biopsy. This relatively high concordance suggests that the model performs well in predicting the initial diagnosis, especially in cases where the clinical features align closely with typical presentations of PV or ET. However, discrepancies in cases like BMB114 and BMB40, where histology indicated an overlap or borderline condition (pre-PMF-/ET-LIKE), highlight the challenges the model faces in classifying more ambiguous cases. When comparing the model’s predictions to the clinical follow-up diagnosis (excluding cases marked as “NA” and unchanged), four of the eight available cases show agreement between the model’s prediction and the final clinical outcome. This corresponds to an 50.0% concordance rate with the follow-up diagnosis. The model’s ability to anticipate the final diagnosis, even in cases where the histology at the time of biopsy was non-specific, underscores its potential to capture subtle disease features that may emerge over time.

**Table 2:** Not otherwise specified (NOS) patients, including their ID codes, the class labels assigned by the model, the scores for each class label, the classifications made by the pathologist at the time of biopsy, and the follow-up classifications.

ID	Prediction	Score pre-PMF	Score PV	Score ET	Histology (timing 0)	Clinical (follow-up)
BMB 103	PV	0.13	0.76	0.11	pre-PMF-/PV-LIKE	PV
BMB 114	ET	0.3	0.35	0.35	pre-PMF-/ET LIKE	UNCHANGED (>LDH)
BMB 15	ET	0.25	0.29	0.47	ET-LIKE	PV
BMB 2	PV	0.18	0.65	0.16	pre-PMF-/ET-LIKE	ET
BMB 24	ET	0.25	0.03	0.72	pre-PMF-/ET-LIKE	UNCHANGED (>LDH)
BMB 36	PV	0.1	0.82	0.08	pre-PMF-/PV-LIKE	UNCHANGED
BMB 40	PV	0.18	0.62	0.2	PV-LIKE	NA
BMB 41	PV	0.18	0.58	0.24	pre-PMF-/ET-LIKE	NA
BMB 63	PV	0.12	0.76	0.12	PV-LIKE	PV
BMB 79	ET	0.27	0.24	0.49	ET-LIKE	ET
BMB 93	ET	0.22	0.15	0.63	ET-LIKE	PV
BMB 1643	ET	0.22	0.04	0.74	ET-LIKE	PMF
BMB 1644	ET	0.22	0.03	0.75	ET-LIKE	ET

The acronym NA stands for Not available.

## Discussion

MPNs present a significant diagnostic challenge due to their heterogeneous nature and overlapping clinical features. Accurate classification of MPN subtypes is crucial for effective patient management and treatment planning. Over the last twenty years, the WHO classifications have progressively lowered the hemoglobin and hematocrit thresholds for PV and the platelet cutoff for ET to enhance the diagnosis of MPNs. However, when strict WHO criteria are applied, about 5% of all MPNs tend to fall into MPN-NOS category (7, 23–26). Most cases diagnosed as MPN-NOS represent a prodromal or pre-proliferative phase of MPNs or early-phase MPNs. Consequently, these cases may share some classical MPN clinical features but do not yet meet the diagnostic criteria required for accurate subclassification (24). Of the 101 patients included in this study, 13 cases (12.9%) were classified as MPN-NOS, as their clinical and pathological features did not meet the diagnostic criteria for any other WHO-defined MPN entity. In our study, 4 out of 13 (30%) cases were reclassified as PV, 1 out of 13 (7.6%) as pre-PMF, and 1 out of 13 (7.6%) as ET during follow-up. The remaining 7 out of 13 (53.8%) cases continued to fall into the MPN-NOS category. Ultimately, six cases were reclassified during follow-up, reducing the rate of MPN-NOS to 6.9% (*see***Table 2**). Frequently, Hb levels, white blood cell (WBC) counts, and LDH levels were normal, while the median platelet count for the entire series was elevated. Notably, the majority of cases demonstrated marrow hypercellularity, normoblastic erythropoiesis, expanded and left-shifted granulopoiesis, and increased numbers of megakaryocytes with loose clustering. Frequently, the megakaryocytes displayed giant forms, hyperlobulated or bulbous nuclei. The most common driver mutation was JAK2, followed by CALR, while no MPL mutations were detected. The patients did not have a history of thrombosis, hemorrhage, or extreme thrombocytosis. Literature studies and our own data confirm the heterogeneity of the MPN-NOS spectrum (27). The clinical presentation of MPN-NOS is variable, with most cases falling within the early, pre-fibrotic stage. It may exhibit increased blood cell counts (thrombocytosis, leukocytosis, and/or erythrocytosis), usually without significant splenomegaly or hepatomegaly. The most common driver mutation is JAK2, followed by CALR and MPL (28). Moreover, the evolution and prognosis of MPN-NOS are variable. In some cases, as observed in our study, follow-up allows for reclassification within a specific MPN, and the prognosis aligns with that of the identified disease. Patients in the early phases tend to have a relatively favourable prognosis, similar to that of ET or pre-PMF. Overall, these studies and our findings suggest that MPN-NOS represents a distinct subgroup within the overall heterogeneous category of patients with MPN-NOS (26). The aims of this study were to further develop AI-based differentiation with a dataset comprising MPN-diagnosed patients. We used MPN-NOS cases in the evaluation of the application of the model. Our model performed well in a moderate multiclass AUC of 0.70 ± 0.01 showing reasonable differentiation between three main MPN subtypes. Our model performed reasonably well for the MPN-NOS cases tested in our model evaluation. The model sometimes rightly predicted the final diagnosis and showed high concordance with the initial diagnosis by pathologists and follow-up by clinicians. Recent studies have explored deep-learning–based approaches for AI-assisted pathological diagnosis of MPNs. In particular, a recent work by (15) employed convolutional neural networks and attention mechanisms applied directly to whole-slide images to capture megakaryocyte phenotypes and spatial context for MPN classification. While such deep-learning approaches have demonstrated strong performance on large datasets, they typically require extensive training data and offer limited interpretability. In contrast, our approach is based on handcrafted, interpretable morphological features extracted from curated ROIs and is therefore particularly suited to small, diagnostically ambiguous cohorts such as early-phase MPNs and MPN-NOS cases. Importantly, both approaches converge on megakaryocyte nuclear morphology as a key discriminative feature, reinforcing the biological relevance of this parameter across different modelling strategies. Discrepancies between model predictions and clinical or histological classifications were mainly observed in cases with overlapping or evolving disease features. For instance, patient BMB114 was classified as ET; however, this case was borderline between pre-PMF and ET, which was evidenced by an increased LDH level. This is a good example of the challenge entailed in the diagnosis of early stages or overlapping conditions in MPNs. Another intricacy in this regard was the case for BMB2 which came into PV according to model classification, but initial pathology showed features overlapping ET and pre-PMF as diagnosis was revised to ET after further discovery of other neoplastic disorders. It would, therefore, be indicative that complex or atypical cases feature clinical follow-up and comprehensive diagnostic workups in MPN-NOS cases. The other complex case was BMB36, for which the model predictions indicated PV, contrasting with the diagnosis of clinical follow-up that was complicated by coexistent thalassemia. This could mask the true pattern of progression for MPNs and hence influence the diagnostic accuracy for this condition. For the rest, for example, BMB15, BMB41, and BMB40, the morphological predictions according to the model did not agree, at least not fully, with the clinical findings. BMB15 was matched mainly as ET; on follow-up, it was seen that this patient had PV, which well illustrates a course for MPNs, often being unpredictable. BMB41 showed features suspicious for PV; however, owing to ambiguity in the clinical data and without any follow-up, it could not be confirmed. Once again, the limited follow-up greatly hampered PV prediction by BMB40 and brought incomplete clinical information into focus. Indeed, in many cases where ET was differentiated from other subtypes, as in the case of the patient BMB93, BMB79, and BMB1644, predictions agreed with final diagnoses during the follow-up and thus reinforced the model capacity for correct identification in consistent presentations. The model has examined the initial phase of illness but cannot foresee the clinical course of a disease. The limitation arises because of complexity in the timeline of the biological history of the disease. Variations among cases that show features of both overlap and evolution signify further refinement of the model. Further studies will involve advanced hyperparameter tuning, training of more diverse and extended datasets in that regard, hoping to improve on this result, especially in complicating situations. In future studies, additional histochemical stains, such as Gomori, will be incorporated to improve the characterisation of stromal and fibrotic components and to further enrich the morphological feature space. Moreover, we plan to systematically compare the current morphology-based machine-learning approach with state-of-the-art deep-learning methods, including convolutional neural networks applied directly to whole-slide images of bone marrow biopsies, in order to evaluate potential performance gains as well as complementary strengths between the two strategies. With regard to ROI selection, regions were defined by two expert pathologists according to shared morphological criteria; however, inter-observer variability was not formally quantified. This limitation will be addressed in future work through a dedicated assessment of inter-observer agreement and by exploring automated ROI detection approaches, with the aim of reducing operator dependence and improving model robustness and reproducibility. Importantly, these developments are planned within the framework of a multicentric study, which will allow evaluation of variability not only in ROI annotation but also across staining protocols, slide scanning technologies, and diagnostic practices. Such an extension will be essential to assess the generalisability of the proposed approach and its applicability in heterogeneous real-world clinical settings.

These would be very important in giving the model more pathological and clinical utilities supporting the management of patients and treatment planning more precisely.

## Conclusion

In summary, this study confirms that bone marrow represents an important component of the diagnostic workup in patients with MPNs. Importantly, the findings of this study support a role for AI-based models to complement pathological diagnosis and clinical decision-making in these often-complex hematological disorders. This model may be the first-building block to define a tool useful for early insight into the classification of disease and its course, thus acting as a valuable adjunct to traditionally diagnostic approaches.

## Data Availability

The raw data supporting the conclusions of this article will be made available by the authors, without undue reservation.
